# Aerosol immunisation for TB: matching route of vaccination to route of infection

**DOI:** 10.1093/trstmh/tru206

**Published:** 2015-01-30

**Authors:** Zita-Rose Manjaly Thomas, Helen McShane

**Affiliations:** The Jenner Institute, Old Road Campus Research Building, Roosevelt Drive, Oxford, OX3 7DQ, UK

**Keywords:** Aerosol, Tuberculosis, Vaccination, Vaccines

## Abstract

TB remains a very significant global health burden. There is an urgent need for better tools for TB control, which include an effective vaccine. Bacillus Calmette–Guérin (BCG), the currently licensed vaccine, confers highly variable protection against pulmonary TB, the main source of TB transmission. Replacing BCG completely or boosting BCG with another vaccine are the two current strategies for TB vaccine development. Delivering a vaccine by aerosol represents a way to match the route of vaccination to the route of infection. This route of immunisation offers not only the scientific advantage of delivering the vaccine directly to the respiratory mucosa, but also practical and logistical advantages. This review summarises the state of current TB vaccine candidates in the pipeline, reviews current progress in aerosol administration of vaccines in general and evaluates the potential for TB vaccine candidates to be administered by the aerosol route.

## Tuberculosis: a global health problem

TB remains a significant global health problem.^1^ Worldwide, in 2012, there were 8.6 million new cases and an estimated 1.3 million deaths, including 320 000 deaths among people infected with HIV.^1–3^ In addition, an estimated one third of the world's population is thought to be latently infected with *Mycobacterium tuberculosis* (*M.tb*), which can reactivate, leading to TB disease. This latently infected population represents a large potential reservoir of TB that is often undiagnosed. There is a 10% lifetime risk of reactivation of latent infection, which increases to a 10% annual risk in people who are co-infected with HIV.^4^ This increased risk is only partially abrogated by anti-retroviral therapy (ART). HIV and TB form a co-epidemic and one of the top priorities as defined by the WHO Global Tuberculosis Report 2013 is to increase ART coverage for HIV-positive, *M.tb*-infected patients, to reduce the risks of reactivation. The scale of the TB disease burden is further compounded by diagnostic challenges, and the emergence of multi, extensively and now totally drug resistant strains of *M.tb*.^1,2,5,6^ Less than a quarter of those estimated to have MDR-TB were diagnosed in in 2012.^1^ The rate of decline of TB incidence worldwide remains slow at approximately 2% per year.^1^ Among the 22 high TB-burden countries, half of them are not on track to reduce incidence, prevalence and mortality in line with targets. This is in part due to resource constraints, ongoing conflicts and the HIV epidemic.^1^ All efforts to control TB are aimed at improved diagnosis, treatment and vaccination.

## TB vaccine strategies

The only licensed vaccine against TB is bacille Calmette-Guérin (BCG), which was developed after 13 years of continuous in vitro passage of *Mycobacterium bovis,* the pathogen that causes TB in cattle.^7^ BCG has been included in the Expanded Programme on Immunisation since 1974. Recent guidelines specify that BCG is not recommended in HIV-infected children or HIV-exposed infants until the HIV status is known, due to the risk of disseminated BCG disease.^8^ When administered at birth, BCG is effective in protecting against disseminated paediatric TB.^1,9,10^ However, the protection conferred by BCG against pulmonary TB in adults is highly variable.^11,12^ Revaccination with BCG provides no substantial additional protection and might be associated with an increased frequency of adverse events.^13,14^

A safer and more effective vaccine that will provide robust protection against pulmonary TB is urgently needed. A strong cell-mediated immune response is essential for protective immunity against TB.^15–18^ Class-II restricted CD4+ T cells, together with the antigen specific release of interferon-gamma (IFNγ) and tumour necrosis factor-alpha (TNFα) are necessary for protection^19–24^ and loss of CD4+ T cells increases the likelihood of succumbing to TB.^25^ IL-2 is important for central memory T cell responses^26^ and Class I restricted CD8+ T cells are also necessary for optimal protection.^27,28^ However, none of these immunological parameters alone correlate well with protection. As there is currently no correlate of protection pre-clinical animal models and in vitro mycobacterial growth inhibition assays have been used as surrogate indicators to guide vaccine selection.^29^ Ultimately, expensive large efficacy trials are required to test vaccine efficacy and advance our understanding about correlates of risk and protection.^30^

In the last decade several candidate vaccines designed to improve immunity against TB have been evaluated in clinical trials.^29^ There are two main approaches. The first is to develop novel whole mycobacterial vaccines that are designed to replace BCG. Two BCG replacement vaccines currently being evaluated in the clinic are MTBVAC and rBCG VPM1002.^31–33^ MTBVAC, an attenuated strain of *M.tb* that has deletions in the genes encoding phoP and fadD26 (both genes involved in *M.tb* virulence) is currently being evaluated in a phase I trial.^31^ VPM 1002 is a live recombinant BCG strain that expresses listeriolysin and has had the urease gene deleted. Listeriolysin perforates the phagosomal membrane and the urease deletion ensures optimum pH for listeriolysin activity.^32^ The rationale for this design is to enhance release of BCG-derived antigens into the cytosol and enhance MHC I presentation of these antigens.^32^ This BCG is more immunogenic and safer than the wild type BCG in mice.^32^ Phase I trials in Germany and South Africa have recently been completed and a Phase IIa trial is underway.^33^

Several prophylactic subunit vaccines have been designed as booster vaccines in BCG-vaccinated individuals or as an alternative vaccine in those for whom live vaccines are contraindicated. Heterologous prime-boost immunisation regimens, where two different vaccines expressing common antigens are given weeks apart, are an effective way to induce strong cellular immune responses.^34–38^ BCG would be the logical priming vaccine in such a prime-boost regimen, to retain the protective effects in childhood. The main categories of subunit vaccines are adjuvanted recombinant proteins or recombinant viral vectors, both inducing immune responses to one or a few selected mycobacterial antigens e.g., antigen 85A/B, ESAT6 and TB10.4. There is some interest in so-called latency antigens such as Rv2660c, particularly for post-exposure vaccines administered to latently infected people to prevent the risk of reactivation. Protein and adjuvant vaccines that have completed phase I trials include Hybrid 1(H1), a fusion protein consisting of antigen 85B fused to ESAT 6, H56, consisting of antigen 85B, ESAT6 and Rv2660c, and HyVaC 4, consisting of Ag85B and TB10.4. All these fusion proteins are being evaluated in clinical trials with IC31, a novel adjuvant that is an activator of Toll-like receptor 9.^39–41^ M72, another fusion protein consisting of 32 kDa and 39 kDa proteins, used with the GSK adjuvant AS01, has been evaluated in several phase I/IIa trials and has recently entered phase IIb efficacy testing (ClinicalTrials.gov Identifier: NCT01755598) with expected completion in November 2018.^42,43^ IDRI′s vaccine candidate, ID93+GLA-SE is composed of a recombinant fusion protein of four *M.tb* antigens, Rv2608, Rv3619, Rv3620 and Rv1813, combined with an oil in water adjuvant and has entered phase I clinical trials.^44^

Viral vector vaccines are based on replication deficient virus variants expressing various immunodominant TB antigens. Viral vectors are a very potent and safe way to induce and boost cellular and humoral immunity. They are widely investigated for malaria, TB, HIV and ebola vaccines and to date have appeared to be well tolerated and immunogenic in all human studies. Examples include MVA85A, a Modified Vaccinia virus Ankara expressing antigen 85A; AdHu5Ag85A a recombinant human type 5 adenovirus expressing antigen 85A; and AERAS-402, an Adenovirus Hu35 expressing Ag85A, Ag85B and TB10.4.^45–47^ MVA85A administered intradermally boosts pre-existing BCG-induced immune responses in adults. In a recent efficacy trial in BCG-vaccinated infants, vaccine induced immune responses were much weaker and no significant improvement in efficacy above BCG alone was seen.^30^ AdHu5Ag85A has been shown to be effective at protecting against *M.tb* challenge when administered intranasally in several animal models as a stand-alone vaccine or as a boost to a BCG prime.^48,49^ A phase I study of intramuscular immunisation with AdHu5Ag85A has recently been completed showing the vaccine to be safe, well tolerated and immunogenic in BCG-naïve and BCG-vaccinated healthy volunteers, with more potent immunogenicity in the latter group.^50^

A limitation of all virally-vectored vaccines is pre-existing or ‘de novo’ anti-vector immunity that potentially precludes homologous boosting.^45,51,52^ However, a recent phase I study with AdHu5Ag85A has demonstrated T cell responses despite pre-existing anti-adenovirus immunity.^50^ Therefore, the functional significance of anti-vector immunity is uncertain.

Concerns about potential immunopathology of TB vaccines and the so called Koch phenomenon, especially in people infected with TB, had been a concern in the early stages of vaccine development. However, serial testing in people with increasing mycobacterial burden over the last decade has demonstrated no immunopathology with MVA85A or any other candidate TB vaccine tested to date.^53^

Another concern is the use of TB vaccines in HIV-infected individuals in whom susceptibility to TB is increased. Live vaccines such as MTBVAC and VPM1002 will pose a potential safety issue in HIV-infected individuals and BCG is contraindicated in this population. However, virally vectored vaccines such as MVA85A have been tested and demonstrated to be safe in HIV-infected individuals.^54^

The vaccine candidates being developed vary in the particular delivery system and antigens but to date have almost entirely been administered systemically. There is an increasing focus on a mucosal route of vaccination, in order to match the route of vaccination to route of natural infection.

## Rationale for aerosol vaccination for TB

The primary route of *M.tb* infection is via inhalation of aerosolised droplets containing *M.tb* resulting in a primary infection focus in the lung. Delivering a TB vaccine by aerosol directly to the respiratory mucosa might offer a physiological and immunological advantage. This route of vaccination also offers potential logistic advantages as it does not require highly trained personnel, thus facilitating deployment; and also avoids risks associated with use and disposal of needles and syringes.

Mucosal immunisation via the intranasal route has also been considered. However, this route of immunisation has raised safety concerns after the epidemiological association of facial nerve paralysis (Bell's palsy) following administration of inactivated influenza virosome vaccine (NasalFlu) containing an *Escherichia coli* heat labile toxin adjuvant (ELT), leading to withdrawal of the vaccine.^55^ In addition, two transient cases of facial nerve palsy were reported following administration of nasal subunit vaccines against HIV and TB in two concurrent phase I clinical trials. In these trials, both protein vaccines were administered with the same ELT adjuvant.^56^

The exact cause and pathogenesis of the facial nerve palsy in these cases remain unclear. In the case of NasalFlu, ‘the study suggests a strong association between the inactivated intranasal influenza’ and for the other two cases using the ELT adjuvant intranasally, ‘the individual components for paralysis were not identified’.^55,56^

## Aerosol vaccination for measles and influenza

There are ongoing efforts to evaluate mucosal routes of vaccination for other respiratory pathogens. Pulmonary delivery of measles vaccine has been explored as an option to boost immunity and interrupt transmission by improving herd immunity.^57,58^ Aerosol boosting of measles vaccination was shown to be effective and acceptable;^59^ and evoked a stronger and more durable antibody response than injected measles vaccine.^60^ The device used to generate the aerosolised vaccine was a commercially available nebuliser that generates particles less than 5 µm diameter using an electrically powered compressor (IPI Medical Products, Division of Inhalation Plastics, Chicago, IL, USA). The currently available injectable vaccine is usually administered subcutaneously and coverage reported as 84% in 2013.^61^ Aerosol delivery of the vaccine could further improve coverage due to ease of administration.

In all published comparative clinical trials, aerosolised measles vaccination was equally or more immunogenic than the subcutaneous vaccination in children aged 10 months or older.^57^ Also, administering a booster dose by the aerosol route yields a stronger and more durable antibody titre than when it is given by the subcutaneous route.^60,62,63^ Mass campaigns in South African and Mexican schoolchildren have also demonstrated that the aerosol route of vaccine delivery is not only safe and immunogenic, but also an acceptable and feasible method.^60,64^ Pulmonary delivery of measles vaccine will require re-licensure. Post licensure field trials will be required to demonstrate efficacy of this route.

For seasonal influenza, an intranasally administered live attenuated influenza vaccine (LAIV) has recently been licensed in addition to the injectable trivalent vaccine (TIV). LAIV demonstrated higher efficacy in children than TIV.^65^ LAIV was also associated with a more sustained duration of protection than TIV.^66^

## Mucosal immunisation with TB vaccines: preclinical data

BCG administered to mice, guinea pigs and macaques by intranasal or aerosol delivery confers greater protection than parenteral BCG vaccination against *M.tb* challenge.^67–71^

Two candidate TB vaccines, both recombinant viral vectors, have demonstrated significant efficacy against challenge in preclinical animal models when administered direct to the respiratory mucosa. AdHu5Ag85A, delivered intranasally, conferred significant protection against aerosol *M.tb* challenge when administered either alone or as a mucosal boost in BCG-immunised animals.^48,49,72^ Single intranasal vaccination with AdHu5Ag85A also offered superior protection to cutaneous BCG vaccination alone.^49^ No enhancement in protection above BCG alone was seen when this vaccine was administered intramuscularly in the murine model.^49^ In cattle, endobronchial boosting of BCG vaccinated animals with AdAg85A induced local mucosal and systemic responses that were similar in magnitude to intradermal boosting.^73^ Mucosal delivery of AdAg85A has not yet been evaluated in humans. MVA85A, administered intranasally to BCG-vaccinated mice, also confers significant protection against aerosol *M.tb* challenge.^74^ When administered by aerosol nebuliser to BCG-vaccinated rhesus macaques, MVA85A induces potent mucosal and systemic immunity.^75^ Interestingly, in this non-human primate study, the strongest responses appeared to be in the compartment to which the vaccine had been administered, although the differences were not statistically significant. Anti-MVA IgG antibodies were detected in the serum of the animals vaccinated by the intradermal route, but not in those vaccinated by the aerosol route.

Recently, AERAS-402 has been delivered by aerosol to BCG-vaccinated and naïve rhesus macaques where it showed robust cellular immune response in the lungs, but failed to confer additional protection against *M.tb* challenge.^76^ The intranasal delivery of inert bacterial spores coated with TB antigens, MPT64 and a hybrid protein consisting of alpha-crystalline (Acr) and antigen 85B, has been found to be immunogenic and conferred significant protection against intranasal *M.tb* challenge in mice.^77^

This body of preclinical data suggests that the aerosol route of delivery may be a promising route of immunisation for recombinant viral vectors, and potentially for TB vaccines in general.

## Aerosol vaccination for TB in humans

There is an early report of BCG being delivered by aerosol in a clinical study. Rosenthal et al. reported aerosol nebulisation of BCG in guinea pigs, school children and medical students.^78^ Rates of tuberculin skin test conversion were used as an outcome measure in this study. Although Rosenthal et al. described technical limitations, in this study, BCG administered by aerosol was well tolerated and feasible in the populations studied. There are some safety and regulatory concerns regarding the administration of BCG, a live replicating mycobacterium, by aerosol in human subjects, and BCG by any route remains contraindicated in immunosuppressed individuals.

The first clinical trial to administer a candidate TB vaccine direct to the respiratory mucosa has recently been reported.^79^ Twenty-four BCG-vaccinated healthy UK adults were randomised to receive MVA85A by aerosol or intradermal administration. The aerosol route of delivery was chosen over the intranasal route in this study because the primary aim was to deliver small particles to the distal airways. MVA85A was administered by aerosol in this trial using a mesh nebuliser MicroAIR NE-U22 (Omron Healthcare UK, Ltd., Milton Keynes, UK). This battery operated device was designed for the delivery of bronchodilators and antibiotics and has improved efficiency of drug deposition into the lungs compared with jet nebulisers.^80^ This nebuliser uses ultrasound to push liquid through a fine metal mesh with 6000 tapered holes generating an aerosol mist with consistently sized particles of about 3 µm in diameter.

Safety was comparable between the two groups, and the aerosol route of immunisation was well tolerated. In bronchoalveolar lavage fluid (BALF) samples taken 7 days after immunisation, antigen specific CD4+ T cells were detectable in both groups of subjects, but were significantly stronger in those subjects immunised by aerosol.^79^ Systemic antigen specific CD4+ T cell responses were comparable between the two groups. Antigen 85A-specific CD8+ T cell responses were higher in the BALF than in the blood after both routes of immunisation. In the blood, Ag85A specific CD8+ T cell responses were significantly higher after aerosol administration than after intradermal administration.^79^ Serum antibodies to MVA were detected after intradermal immunisation but not after aerosol administration of the vaccine.^79^

A second study with aerosolised MVA85A is underway where we evaluate the potential utility of boosting with MVA85A by heterologous routes (NCT01954563).

## Future considerations

The aerosol route of delivery offers practical advantages and potential cost benefits, therefore field trials to evaluate new TB vaccines using aerosol administration is important.

A reliable and robust animal model that reflects the human response to vaccination is urgently required. In the murine model of mucosal vaccination, vaccine responses are compartmentalised. In the non-human primate model, responses appear less compartmentalised, but nevertheless magnitude of response seems to be loyal to route, with strongest responses in the systemic circulation after systemic delivery and strongest responses in the BALF in animals immunised by aerosol. In humans, the responses are somewhat different, with stronger responses in the BALF after aerosol administration, equivalently in whole blood after aerosol administration. Importantly, no reduction in systemic responses is seen after aerosol immunisation. Given the limitations in the animal models and the differences in anatomy between the various models, there is merit in evaluating this route of immunisation in humans, in parallel with the animal models. The best evaluation would be a human safety and immunogenicity experiment side-by-side with a parallel NHP challenge experiment.

To date, the focus in TB vaccine development has been on prophylactic vaccine, but there is now increased interest in therapeutic vaccines for those with TB disease and post-exposure vaccination for individuals latently infected with *M.tb,* given the significant global burden of this population, especially in endemic countries. One of the benefits of a postexposure vaccine would be to sterilise dormant *M.tb* bacteria and thereby prevent reactivation and potentially reinfection. The development of a post-exposure vaccine has in the past been overshadowed by concerns in the field about the so called Koch phenomenon, a mycobacteria-specific immunopathology seen when culture filtrate tuberculosis protein was given to patients with TB.^81^ In early clinical vaccine development, vaccines were tested in serial trials in individuals with increasing mycobacterial burden. MVA85A administered by intradermal injection was safely administered systemically to individuals latently infected with *M.tb.*^53^ Equal care will need to be taken to ensure the safety of the aerosol route of immunisation in subjects with latent *M.tb* infection, prior to this route of immunisation being evaluated in TB high-burden countries.

To make the aerosol route of vaccination practical in the field, further work is needed on vaccine formulation. Powdered BCG vaccine for inhalation has been generated by spray drying^82^ and aerosol delivery of BCG nanomicroparticles has been shown to protect guinea pigs from *M.tb* challenge better than parenteral BCG.^69^

In parallel with efforts to evaluate the aerosol route of vaccine delivery, new methods of immunomonitoring for aerosol vaccination need to be explored. Bronchoscopy and bronchoalveolar lavage are likely to represent a robust and representative sample of mucosal immunity, but remain an invasive method. One method that could be explored for immunomonitoring of respiratory mucosal immunity is induced sputum. Induced sputum has been used to assess airway inflammation in asthma, cystic fibrosis and healthy volunteers and also as an immunoassay in patients with tuberculosis.^83–87^ Furthermore, markers in the blood that correlate with established immunity in the respiratory mucosa, and study of mucosal homing receptors would be a useful non-invasive tool for immunosurveillance, especially in those considered high risk.

The first study of a viral vector vaccine administered by aerosol to humans has been successfully completed. Further studies looking at selection of aerosol TB vaccine candidates in representative animal models, studying aerosol delivery of other TB vaccine candidates, developing the aerosol route for use in the field evaluation in TB high-burden countries and identifying robust immunomonitoring techniques will all facilitate the evaluation and development of the aerosol delivery of TB vaccines.
